# Metachronous Second Primary Malignancies after Head and Neck Cancer in a Korean Cohort (1993-2010)

**DOI:** 10.1371/journal.pone.0134160

**Published:** 2015-07-28

**Authors:** Yuh-S Jung, Jiwon Lim, Kyu-Won Jung, Junsun Ryu, Young-Joo Won

**Affiliations:** 1 Specific Organs Cancer Branch, Department of Otolaryngology, Center for Thyroid Cancer, Research Institute and Hospital, National Cancer Center, Goyang, Korea; 2 Cancer Registration and Statistics Branch, Division of Cancer Registration and Surveillance, National Cancer Center, Goyang, Korea; National Health Research Institutes, TAIWAN

## Abstract

Second primary malignancy (SPM) is the major long-term cause of patient mortality with head and neck squamous cell carcinoma (HNSCC). As the incidence of high-risk human papillomavirus (HPV)-related HNSCC is increasing globally, we analyzed the patterns of SPM occurrence, the effect of the index tumor site along with attributes to HPV, and the effect of SPM on survival in South Korean patients with head and neck cancer (HNC). Data were retrieved from the Korea Central Cancer Registry, a nationwide population-based cancer registry, from 1993 to 2010. Standardized incidence ratios were analyzed and compared between index tumor sites, particularly oropharyngeal vs. non-oropharyngeal sites. After adjustment for competing risks, 3- and 5-year SPM rates were calculated using the cumulative incidence function. The effects of SPM occurrence on overall survival (OS) were then analyzed. SPM rates were significantly lower for HPV-attributable oropharyngeal sites than for non-oropharyngeal sites, such as the larynx and hypopharynx (*p*<0.001). SPM rates were also lower for oral cavity first primary sites than for non-oropharyngeal first primary sites (*p*<0.001). SPMs typically occurred in the esophagus, lungs and the head and neck. Uterine cervical cancers occurred significantly more frequently after index oropharyngeal cancer in women. The 5-year and 10-year OS rates were 57.8 and 45.7% in all HNC patients, respectively. The OS after SPM occurrence was poor (5-year, 31.8%; 10-year, 20.8%) compared to after index HNC occurrence (5-year, 68.4%; 10-year, 41.2%). SPM occurrence in the esophagus and lung/bronchus showed a worse OS than SPM localized to the head and neck. South Korean HNC patient, the first primary cancer site affected SPM risk and distribution. The 5- and 10-year OS rates deteriorated after SPM occurrence, particularly in the esophagus and lungs. Further optimization of follow-up strategies for effective surveillance of SPM, particularly in the esophagus and lungs, is warranted.

## Introduction

Second primary malignancy (SPM) is reported as the leading long-term cause of mortality following treatment of head and neck squamous cell carcinoma (HNSCC) in the Western literature [1]. Approximately one-third of HNSCC deaths have been attributed to SPMs [2], which is nearly triple the number of deaths as a result of distant metastases [3]. Thus, although survival rates have shown recent improvements for some HNSCC patients due to the progress in HNSCC treatment, improvements in tobacco control, and the increase in the detection of human papillomavirus (HPV)-associated HNSCCs, synchronous (<6 months after initial HNSCC diagnosis) and metachronous (≥6 months after initial HNSCC diagnosis) SPMs remain a major obstacle for long-term survival in the Western population [4, 5]. However, SPM patterns and risk according to the index HNSCC site (oral cavity, oropharynx, larynx, and hypopharynx) with possible associations with HPV and the prognostic effects of SPM occurrence have not been well-characterized, particularly in Asian countries.

In recent years, high-risk (HR) HPV has replaced tobacco and alcohol exposure as the etiological agent responsible for most HNSCC cases arising in the oropharynx in the US, Europe [6, 7], and Asian countries such as South Korea [8, 9]. In contrast to patients with HNSCCs from other sites, for the majority of patients with oropharyngeal squamous cell carcinomas (SCCs), carcinogenesis is mainly driven by high-risk HPV rather than smoking or alcohol. Therefore, we hypothesized that the SPM risk and distribution may differ between patients with HPV-attributable oropharyngeal SCCs and those with SCCs from HPV-nonattributable sites, such as the larynx and hypopharynx. In support of our hypothesis, an association between SPM risk and location and HPV status in never smokers with index oropharyngeal cancers was recently reported in the US [10]. Although confirmation of this association has not yet been demonstrated in Asian countries such as South Korea, these data support the utility of site-specific screening strategies for SPMs after the treatment of primary cancer. Moreover, because worse survival outcomes in HNSCC patients with an SPM have been documented, detailed survival outcomes according to the type of SPM and subsequent survival outcomes should be additionally investigated to obtain a better clinical picture for such patients.

Given these considerations, we analyzed the site-specific risk of metachronous SPM with an interest in HPV association and its effect on overall survival (OS) after index HNSCC in South Korean patients using data from the comprehensively collected, nationwide population-based Korea Central Cancer Registry (KCCR) for the period from 1993 to 2010.

## Materials and Methods

The KCCR is a nationwide, hospital-based cancer registry that was initiated by the Ministry of Health and Welfare in 1980. The KCCR originally collected information on approximately 80–90% of cancer cases from more than 180 training hospitals across South Korea. In 1999, it was expanded to cover the entire South Korean population under the Population-Based Regional Cancer Registry Program [11]. In the KCCR, cancer sites are classified according to the International Classification of Diseases for Oncology, 3rd edition [12]. According to the International Agency for Research on Cancer rules, a SPM was classified when a new primary tumor had a different anatomical site or histological type from the index cancer. Since a SPM within the first 6 months after diagnosis of the first primary cancer was considered a synchronous cancer, it was then excluded from this study.

The institutional review board at the National Cancer Center approved this study (NCC2015-0133). Using the KCCR, a total of 49,838 head and neck cancer (HNC) cases between 1993 and 2010 were identified. HNC included the following anatomical sites: lips, oral cavity, and pharynx (C00-C14); nasal cavity (C30); accessory sinus (C31); and larynx (C32). Subgroup analyses were performed according to the anatomical location of the primary cancer: HPV-attributable sites (the oropharynx including the tonsils [C09-C10, C14]); HPV-nonattributable sites (the hypopharynx [C12-C13] and the larynx [C32]); and the oral cavity [C00-C06]. HPV-attributable and nonatttibutable sites were based on previously published reports [13]. Standardized incidence ratios (SIRs) and the corresponding 95% confidence intervals (95% CIs) of SPM among patients with HNC were analyzed to quantify the relative risk compared with the general population. SIR was calculated as the ratio of observed numbers to expected numbers of SPMs, which was obtained by assuming that these patients experienced the same cancer incidence as the corresponding general population based on 5-year age, 1-year calendar period, and sex-specific by cancer type. A mid-year population in Korea is available from http://kosis.kr/. The CIs of the SIRs were based on the assumption of a Poisson distribution for second cancer cases. The number of person-years at risk (PYR) was calculated from 6 months after diagnosis of the first primary cancer to the date of death or the end date of this study (December 31. 2010), whichever occurred first. Excess absolute risk (EAR) is an absolute measure of the clinical burden due to SPM. EAR was expressed per 10,000 PYR and calculated by [(observed number-expected number)/PYR]x10,000. We calculated cumulative incidence as the percentage of patients developing a SPM 3 and 5 years after the first primary cancer diagnosis, considering competing risk as death. The cumulative incidence rates (CIRs) were analyzed and compared according to the index cancer site, particularly oropharyngeal vs. non-oropharyngeal sites. Kaplan-Meier survival curves were also calculated for head and neck cancer patients with or without SPM. The differences between groups of CIRs and survival curves were assessed using the log-rank test. A determination of statistical significance was based on two-sided *p*<0.05. To compute SIR and EAR, we used SEER*stat (version 8.1.2; SEER program, National Cancer Institute). CIRs and survival curves were generated, and log rank tests were performed in SAS (version 9.2; SAS Institute) and STATA (version 12.1; StataCorp LP).

## Results

A total of 49,838 patients who were diagnosed with primary HNC were evaluated over a mean follow-up period of 4.46 years. The mean age at initial HNC diagnosis was 57.91 years. Among the patients with HNC, 3,164 patients (6.35%) eventually developed metachronous SPMs. The mean age of occurrence was younger in patients with oropharynx (56.62 years) and oral cavity cancers (58.02 years) than in patients with larynx (62.67 years) and hypopharynx cancers (62.82 years) **(** Table 1 **)**.

**Table 1:** Patient characteristics according to head and neck sites.

		Head and neck^a^		Oral cavity^b^		Oropharynx^c^		Larynx^d^		Hypopharynx^e^	
		No.	%	No.	%	No.	%	No.	%	No.	%
**No. of patients**		49,838	100.00	11,235	100.00	5,587	100.00	15,113	100.00	3,685	100.00
**No. of patients with subsequent cancer**		3,164	6.35	699	6.22	298	5.33	1,306	8.64	240	6.51
**Mean follow-up, years (SD)**		4.46 (4.40)		4.22 (4.38)		4.15 (4.20)		5.04 (4.52)		2.61 (3.36)	
**Mean age at diagnosis, years (SD)**		57.91 (13.94)		58.02 (14.13)		56.62 (13.57)		62.67 (9.96)		62.82 (9.63)	
**Sex**	**Male**	38,516	77.28	7,600	67.65	4,376	78.32	13,954	92.33	3,472	94.22
	**Female**	11,322	22.72	3,635	32.35	1,211	21.68	1,159	7.67	213	5.78
**Age at diagnosis**	**<30**	1,965	3.94	399	3.55	191	3.42	45	0.30	11	0.30
	**30–49**	9,963	19.99	2,444	21.75	1,323	23.68	1,323	8.75	298	8.09
	**50–69**	28,052	56.29	6,036	53.72	3,147	56.33	9,983	66.06	2,480	67.30
	**70–79**	8,179	16.41	1,878	16.72	773	13.84	3,212	21.25	762	20.68
	**>79**	1,679	3.37	478	4.25	153	2.74	550	3.64	134	3.64
**Year at diagnosis**	**1993-19951993-1995**	6,070	12.18	1,328	11.82	567	10.15	2,061	13.64	456	12.37
	**1996–1998**	7,243	14.53	1,594	14.19	715	12.80	2,416	15.99	547	14.84
	**1999–2001**	8,415	16.88	1,819	16.19	852	15.25	2,736	18.10	642	17.42
	**2002–2004**	9,243	18.55	2,089	18.59	1,106	19.80	2,739	18.12	718	19.48
	**2005–2007**	9,913	19.89	2,283	20.32	1,193	21.35	2,738	18.12	719	19.51
	**2008–2010**	8,954	17.97	2,122	18.89	1,154	20.66	2,423	16.03	603	16.36

^a^C00-C14, C30-C31, C32.

^b^C00-C06.

^c^C09-C10, C14.

^d^C32.

^e^C12-C13.

Metachronous SPMs typically occurred in the head and neck, esophagus, lung, and thyroid (Table 2) after cancers of all primary sites in the head and neck, which includes cancers of the oral cavity, oropharynx, larynx, and hypopharynx. SPMs occurred significantly more frequently in head and neck sites after cancers of the oral cavity, oropharynx, larynx, and hypopharynx than other sites. Among organs outside the head and neck, esophageal cancers most frequently occurred after HNC (SIR, 4.60; 95% CI, 4.08–5.17). Of note, esophageal cancers occurred significantly more frequently after cancer of the hypopharynx (SIR, 17.75; 95% CI, 13.86–22.39). Lung cancers also occurred significantly more frequently after HNC (SIR, 2.01; 95% CI, 1.88–2.15). Interestingly, in women, the incidence of secondary uterine cervical cancers was significantly higher after index oropharyngeal cancer (SIR, 3.11; 95% CI, 1.14–6.77) than after index non-oropharyngeal cancer (data not shown).

**Table 2:** Risk of SPM according to head and neck sites.

	Head and neck^a^			Oral cavity^b^			Oropharynx^c^			Larynx^d^			Hypopharynx^e^		
SPM site	SIR	95% CI	EAR	SIR	95% CI	EAR	SIR	95% CI	EAR	SIR	95% CI	EAR	SIR	95% CI	EAR
**Total**	1.40*	1.35–1.45	44.04	1.58*	1.47–1.69	57.68	1.46*	1.3–1.63	43.36	1.26*	1.19–1.32	38.08	1.93*	1.71–2.17	135.64
**Head and neck**	6.01*	5.5–6.55	19.19	10.27*	8.74–11.99	30.46	9.00*	6.99–11.4	26.08	1.90*	1.51–2.37	4.92	9.14*	6.74–12.12	44.45
Oral cavity	10.01*	8.58–11.61	7.08	19.57*	15.11–24.95	13.01	22.29*	15.52–31	14.42	3.53*	2.36–5.06	2.73	10.61*	5.3–18.99	10.36
Oropharynx	5.38*	4.01–7.08	1.87	13.22*	8.38–19.84	4.48	3.42	0.7–9.99	0.92	2.22*	1.07–4.09	0.72	0	0–6.5	-0.59
Larynx	2.86*	2.3–3.51	2.66	4.11*	2.6–6.16	3.67	4.79*	2.55–8.19	4.44	0.31*	0.1–0.72	-1.48	15.47*	10.58–21.84	31.13
Hypopharynx	4.80*	3.55–6.35	1.74	10.66*	6.42–16.64	3.63	0	0–4.27	-0.37	4.56*	2.92–6.79	2.46	1.5	0.04–8.37	0.35
**Esophagus**	4.60*	4.08–5.17	9.89	5.87*	4.51–7.51	11.03	5.67*	3.8–8.14	10.31	3.42*	2.81–4.13	10.03	17.75*	13.86–22.39	69.67
**Lung**	2.01*	1.88–2.15	19.29	1.91*	1.62–2.25	15	1.69*	1.29–2.18	10.6	2.15*	1.96–2.36	32.21	2.33*	1.79–2.99	36.86
**Stomach**	0.90*	0.82–0.98	-2.37	0.95	0.77–1.17	-0.97	0.83	0.59–1.14	-3.26	0.92	0.8–1.05	-2.53	1.03	0.7–1.46	0.8
**Colon**	0.87	0.74–1.02	-1.01	0.68	0.44–1.01	-2.36	0.86	0.47–1.44	-1	0.95	0.75–1.19	-0.49	0.89	0.41–1.69	-1.17
**Liver**	0.92	0.8–1.06	-0.77	1.02	0.73–1.38	0.18	1.42	0.95–2.04	3.72	0.89	0.71–1.09	-1.47	0.89	0.44–1.58	-1.48
**Thyroid**	1.41*	1.15–1.71	1.33	1.24	0.79–1.87	0.95	1.06	0.49–2.02	0.23	1.80**	1.19–2.62	1.58	0.5	0.01–2.76	-1.06
**Anogenital**	0.93	0.63–1.32	-0.11	0.68	0.28–1.41	-0.68	1.62	0.6–3.53	0.99	0.81	0.22–2.09	-0.12	0	0–6.54	-0.59
Cervix	1.15	0.7–1.78	0.12	0.55	0.11–1.6	-0.52	3.11*	1.14–6.77	1.76	0.47	0.01–2.63	-0.15	0	0–16.36	-0.23
Other anogenital	0.68	0.34–1.22	-0.23	0.84	0.23–2.15	-0.16	0	0–2.08	-0.76	1.07	0.22–3.14	0.03	0	0–10.9	-0.35

SPM: Second primary malignancy, CI: Confidence interval, SIR: Standardized incidence ratio, EAR: Excess absolute risk per 10,000 person-years.

^*^significant at alpha = 0.05

^a^C00-C14, C30-C31, C32.

^b^C00-C06.

^c^C09-C10, C14.

^d^C32.

^e^C12-C13.

**Fig 1:**
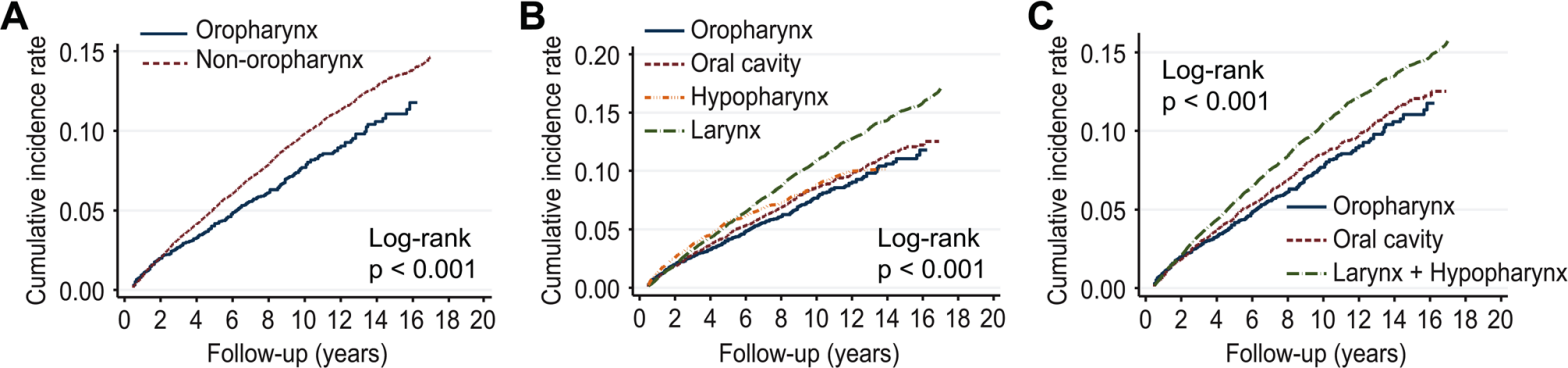
Cumulative incidence function of SPMs according to the primary cancer site, considering competing risks. A. Oropharynx and non-oropharynx. B. Oropharynx, oral cavity, larynx and hypopharynx. C. Oropharynx, oral cavity, larynx + hypopharynx (L+H).

**Fig 2:**
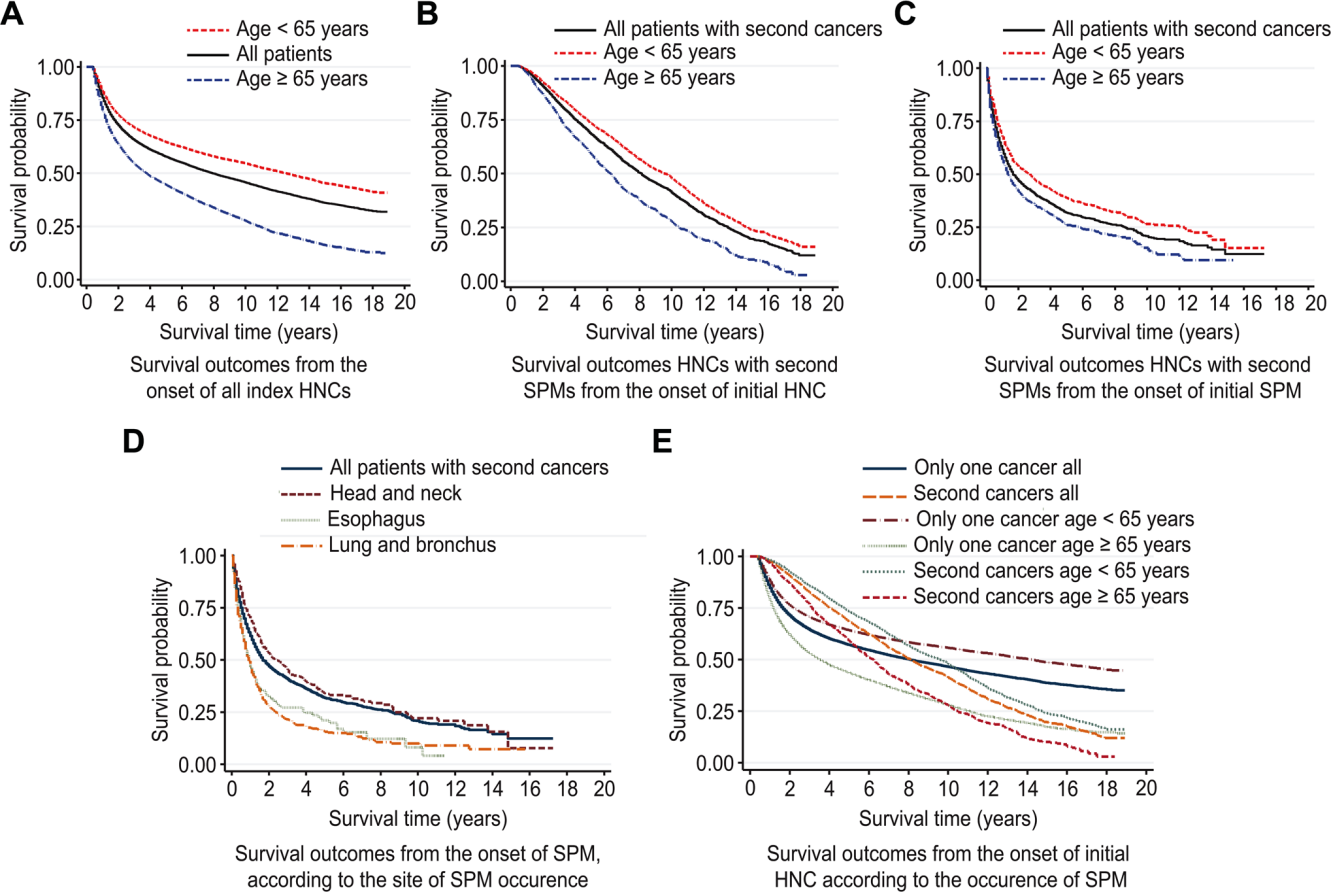
Survival outcomes from the onset of all index HNCs (A). Survival outcomes HNCs with second SPMs, from the onset of initial HNC(B) and from the onset of SPM (C) according to the age (age <65 versus age ≥65). Survival outcomes from the onset of SPM, according to the site of SPM occurrence (D). Survival outcomes from the onset of initial HNC according to the occurrence of SPM (E).

The CIRs of metachronous SPM by the first primary cancer site are shown in Table 3. SPMs occurred more frequently in patients with laryngeal and hypopharyngeal cancers than in those with oral cavity or oropharyngeal cancers. The 3-year and 5-year metachronous SPM rates were significantly lower for oral cavity first primary sites (3-year SPM rate, 2.78 [95% CI, 2.47–3.11]; 5-year SPM rate, 4.44 [95% CI, 4.03–4.88]) and oropharyngeal (3-year SPM rate, 2.66 [95% CI, 2.24–3.14]; 5-year SPM rate, 4.02 [95% CI, 3.47–4.62]) than for non-oropharyngeal first primary sites (3-year and 5-year SPM rates, 3.14[95% CI, 2.93–3.35] and 5.12[95% CI, 4.85–5.40], respectively [data not shown]) such as the larynx (3-year SPM rate, 3.27 [95% CI, 2.98–3.58]; 5-year SPM rate, 5.49 [95% CI, 5.10–5.90]) and hypopharynx (3-year SPM rate, 3.70 [95% CI, 3.10–4.38]; 5-year SPM rate, 5.67 [95% CI, 4.89–6.53]) (Table 3). Esophageal cancers occurred more frequently after hypopharynx cancer (3-year SPM rate, 0.98 [95% CI, 0.69–1.36]; 5-year SPM rate, 1.51 [95% CI, 1.13–2.00]). Lung cancers occurred more frequently after larynx cancer (3-year SPM rate, 1.07 [95% CI, 0.91–1.26]; 5-year SPM rate, 1.84 [95% CI, 1.61–2.09]) and tended to occur more frequently after hypopharynx cancer (3-year SPM rate, 0.92 [95% CI, 0.64–1.30]; 5-year SPM rate, 1.42 [95% CI, 1.04–1.90]). In addition, stomach cancer tended to occur more frequently after cancers of the larynx and hypopharynx than cancers of the oropharynx and oral cavity, which interestingly did not show as steep an incidence rate as esophageal cancer. The cumulative SPM incidence was significantly higher for non-oropharyngeal first primary sites compared to oropharyngeal first primary sites (Fig 1A). The cumulative SPM incidence was also significantly greater for laryngeal and hypopharyngeal first primary sites compared to oropharyngeal and oral cavity sites (Fig 1B and 1C).The 5-year and 10-year OS rates from the onset of the index HNC (Fig 2A) were 57.8 and 45.7% in all patients with HNC, respectively. OS was consistently affected by age (age<65 years: 5-year OS, 64.7%, 10-year OS, 54.6%; age≥65 years: 5-year OS, 44.5%, 10-year OS, 27.6%, *p*<0.05) in groups with or without SPM after the index HNC (Fig 2E). Survival was severely hampered once SPM occurred. The OS after SPM occurrence was dismal (5-year, 31.8%; 10-year, 20.8%, Fig 2C) compared to that after index HNC occurrence (5-year, 68.4%; 10-year, 41.2%, Fig 2B). Notably, the site of SPM occurrence also significantly affected OS (Fig 2D). SPM occurrence in the esophagus and lung/bronchus was associated with worse OS than SPM localized within the head and neck. The effect of age and the site of SPM occurrence on OS was consistent when we analyzed the outcome after individual index HNC sites, such as the oral cavity, larynx, and hypopharynx.

**Table 3:** Cumulative incidence rate(%) of SPM according to head and neck sites.

	Oral cavity^a^		Oropharynx^b^		Larynx^c^		Hypopharynx^d^	
SPM site	3-year(95% CI)	5-year(95% CI)	3-year(95% CI)	5-year(95% CI)	3-year(95% CI)	5-year(95% CI)	3-year(95% CI)	5-year(95% CI)
**Total**	2.78(2.47–3.11)	4.44(4.03–4.88)	2.66(2.24–3.14)	4.02(3.47–4.62) *	3.27(2.98–3.58)	5.49(5.10–5.90)	3.70(3.10–4.38)	5.67(4.89–6.53)
**Head and Neck**	0.77(0.61–0.95)	1.04(0.85–1.23) *	0.82(0.60–1.10)	1.11(0.84–1.45) *	0.27(0.19–0.37)	0.33(0.24–0.44)	0.86(0.59–1.22)	1.24(0.89–1.67)
Oral cavity	0.35(0.25–0.48)	0.44(0.33–0.59)	0.47(0.30–0.69)	0.60(0.41–0.86) **	0.06(0.03–0.11)	0.09(0.05–0.15)	0.06(0.01–0.22)	0.18(0.07–0.40)
Oropharynx	0.09(0.05–0.18) *	0.18(0.11–0.28)	0.02(0.00–0.11)	0.04(0.01–0.16)	0.04(0.02–0.09)	0.05(0.02–0.10)	-	-
Larynx	0.10(0.05–0.18) *	0.14(0.08–0.24)	0.15(0.07–0.30)	0.18(0.09–0.33)	0.02(0.01–0.06)	0.03(0.01–0.08)	0.68(0.45–1.01)	0.91(0.62–1.29)
Hypopharynx	0.07(0.03–0.15)	0.09(0.04–0.17)	-	-	0.11(0.06–0.18) *	0.12(0.07–0.19)	0.03(0.00–0.19) *	0.03(0.00–0.19) *
**Esophagus**	0.25(0.17–0.37)	0.39(0.28–0.54)	0.18(0.09–0.34)	0.34(0.20–0.56)	0.26(0.18–0.36)	0.41(0.31–0.54)	0.98(0.69–1.36)	1.51(1.13–2.00)
**Lung**	0.49(0.37–0.64)	0.84(0.67–1.05) *	0.41(0.26–0.62)	0.84(0.60–1.16)	1.07(0.91–1.26)	1.84(1.61–2.09) *	0.92(0.64–1.30)	1.42(1.04–1.90)
**Stomach**	0.27(0.18–0.39)	0.44(0.32–0.59)	0.18(0.09–0.34)	0.32(0.18–0.53)	0.54(0.43–0.68)	0.89(0.74–1.07))	0.37(0.20–0.64)	0.56(0.34–0.89)
**Colon**	0.09(0.05–0.17)	0.16(0.09–0.26)	0.12(0.05–0.25)	0.14(0.06–0.29)	0.15(0.09–0.23)	0.26(0.18–0.36)	0.09(0.03–0.25)	0.12(0.04–0.31)
**Liver**	0.09(0.05–0.17)	0.25(0.16–0.37)	0.23(0.12–0.40)	0.37(0.22–0.59)	0.18(0.12–0.26)	0.42(0.32–0.55)	0.09(0.03–0.26)	0.17(0.06–0.38)
**Thyroid**	0.09(0.05–0.17)	0.13(0.07–0.22)	0.14(0.06–0.28)	0.14(0.06–0.28)	0.12(0.08–0.19)	0.13(0.08–0.21)	-	-
**Cervix**	0.01(0.00–0.06) *	0.02(0.01–0.08)	0.08(0.03–0.20)	0.11(0.04–0.25)	0	0.01(0.00–0.06)	-	-

*significant at alpha = 0.05

^a^C00-C06.

^b^C09-C10, C14.

^c^C32.

^d^C12-C13.

## Discussion

Because SPMs have become recognized as a leading long-term cause of poor survival in patients with HNSCC in the Western population [14], we sought to describe the site-specific patterns of SPM after index HNSCC occurrence. A few Western studies have reported that SPM risk and location are significantly associated with the primary HNSCC site [4, 15]. These studies have implied that the possible conflicting carcinogenetic mechanisms of tobacco smoke exposure and HR-HPV, which have emerged in recent decades, may influence SPM risk and location [16, 17]. However, little is known about these factors in Asian populations. Herein, we analyzed the SPM risk and distribution in South Korean HNSCC patients using a nationwide population-based database. Our study provides a comprehensive picture of SPM occurrence following index HNCs and thus has important implications for further analyses of SPM patterns, risks in HNCs and eventual prospects for future changes.

Because metachronous SPMs occur at a higher frequency than synchronous SPMs [14, 18, 19], we focused on metachronous SPMs after primary HNC to alleviate the possible bias of population-based registry data during the initial diagnostic process and subsequent cancer registration process. Of all the patients diagnosed with first primary oropharyngeal cancer, only 2.66% of patients harbored an excess metachronous SPM within 3 years. This result implies that first primary oropharyngeal cancers, which are known to be HPV-attributable cancers, are associated with a lower SPM rate compared to first primary non-oropharyngeal cancers, which are known to be HPV-nonattributable cancers. Our data are in line with Western data that have demonstrated a lower SPM risk for HPV-attributable HNSCCs than for HPV-nonattributable HNSCCs [4]. Overall, the SPM prevalence in Korea was 6.35%, which is comparable to or on the high margin of the estimated SPM prevalence of 1–6% in Western countries, indicating the need for further effective surveillance strategies in Korea [15, 20]. Recent population-based data of 1,658 patients with primary HNC from British Columbia (BC) showed that the actual metachronous SPM incidence was 15.1% at 5 years, eventually reaching 61% at 25 years [21]. This incidence is higher than our incidence of 6.35% at 5 years. Overall, longer follow-up is needed for the eventual parallel comparison between these two population groups.

Because the rate of SPM occurrence after HNC at oropharynx sites compared to other sites has uniquely declined in the US over the last 30 years [22], we initially expected similar changes in South Korea. Although these US data focused on synchronous SPM occurrence, which might not be comparable to our metachronous SPM rates, we failed to confirm a concordant decrease of SPM after index oropharyngeal cancer in our Korean cohort. This chronological discordance of the SPM pattern after HNC between the US and Korea may be due to the relatively short-term follow-up periods and smaller case numbers in the Korean cohort, which may not be sufficient to observe differences in absolute SPM rates. Another explanation is that the increase in HPV-attributable HNCs in Korea may not yet be as steep as that in Europe and the US. Although our previous report on nationwide changes in HNC occurrence showed a gradual increase in oropharyngeal cancers and a decrease in laryngeal and hypopharyngeal cancers in Korea [9], the annual percent change in oropharyngeal cancer remained lower in Korea than in the US and Europe. This finding implies that the HPV epidemic may be occurring more gradually in Korea, resulting in a more gradual change in SPM occurrence. This gradual effect may be attributed to the fact that South Korea is a recently developed country with a less Westernized and more traditional culture, particularly in terms of smoking and sexual behavior preferences. However, additional population-based studies should be designed to confirm these speculations.

Our results also showed that cancers of the esophagus (SIR, 4.60) and lungs (SIR, 2.01) were the main sites of occurrence of metachronous SPM outside of the head and neck area. These results are in line with recent data from the BC population group [21]. This result implies that HPV-nonattributable cancers might occur through carcinogenetic mechanisms, inflicting the entire dimension of the upper aerodigestive tract (UADT). Notably, hypopharyngeal cancers carried the highest SPM risk of any HNC sites in the Korean population. Furthermore, our data consistently confirmed that the occurrence of SPM significantly affected survival, which is comparable to data from Western populations [4, 5]. OS was significantly worsened between 5 and 10 years after index HNC, particularly after larynx and hypopharynx index primary sites. Similar to previous results [21], occurrence of SPM in the lung and esophagus reduced the overall survival in our study. Age (65 years) also affected the survival of HNC at all sites, either with or without SPMs. Interestingly, SPM occurrence in the esophagus and lung/bronchus was associated with worse OS than SPM localized within the head and neck.

Because our data indicated that cancers of the larynx and hypopharynx were associated with an increased risk of metachronous SPM in the esophagus and lung and that these situations mostly lead to dismal outcomes, Asian populations with index larynx and hypopharynx cancers are likely to benefit from additional careful surveillance strategies such as regular esophagoscopy for the early detection of possible SPMs and prevention of poor outcomes [23]. Similarly, lung cancers developed more frequently from cancers of all head and neck sites, particularly after index larynx and hypopharynx cancers, and this result further shows the importance of careful surveillance strategies, such as screening chest computed tomography (CT) at adequate intervals after the initial treatment of HNC. Stomach cancer also tended to occur more frequently after cancers of the larynx and hypopharynx than cancers of oral cavity and oropharynx, which interestingly did not show as steep an incidence rate as esophageal or lung cancer. We also interpret this result as the increased carcinogenetic pressure from smoking and other hazards that damage the gastric mucosa, albeit to a lesser degree than damage to the esophagus.

These data could also be used to develop specific SPM screening strategies for Asian populations with HNC. Routine follow-ups by head and neck specialists and careful workups on the esophagus, lungs, and stomach might be important for early detection of SPMs and thus improvements in long-term survival in the Korean population.

One interesting result was the increased risk of secondary uterine cervical cancers after index oropharyngeal cancer compared to after index non-oropharyngeal cancer in women. Relationships between cancers of the oropharynx and uterine cervix were reported in the US, Canada [24], and Italy [25], and our Korean data are in line with these relationships. However, considering that this correlation between two HPV-related cancers was not detected in another study [26], further detailed evaluation is warranted.

HPV shows tropism for certain areas bearing tonsillar crypt structures on the UADT; for instance, synchronous HPV-associated tonsillar carcinomas [27] and HPV-associated nasopharyngeal carcinomas have been reported [28]. Furthermore, recent reports have suggested that the field effect, multiple independent HPV infections, and the migration of HPV-infected cells might be potential molecular mechanisms for the development of multifocal HPV-associated HNCs [29]. However, we could not confirm these findings in our cohort, which may have been due to the limitations of our population-based data. Nonetheless, considering that significantly fewer SPMs occur on organs outside the head and neck, such as the esophagus and lungs, after index oropharynx primary cancers, our data still support the finding that HPV-related cancers may harbor different unique carcinogenetic mechanisms and cause less damage to organs outside the head and neck. Nevertheless, the molecular mechanisms involved in SPM development must be further elucidated, particularly in Asian populations.

Our study has some limitations. First, the KCCR does not record cancer risk factors, such as tobacco/alcohol use or HPV status, and this lack of data makes it more difficult to provide a real picture for the relationship between these etiological factors and SPM risk. In addition, the detailed features of SPMs, particularly of the head and neck, were occasionally unclear or not available for our analysis. Nevertheless, we believe that the quality controls and high-quality multiple primary data of the KCCR, which contains information that is over 97% complete and encompasses the entire South Korean population [30], might allow for the analysis of excess risks with excellent internal validity and generalizability, enough for the speculations in this study.

Our findings demonstrate that HNC patients have a markedly escalated SPM risk, and this risk is influenced by the characteristics of the patients and tumors, particularly the primary tumor site. Certain subsets of HNC patients may benefit from the rational application of various screening modalities. Thus, our hypothesis-driven study may potentially be useful for further optimization of SPM detection strategies in this high-risk population.
